# An Uncommon Encounter: A Comprehensive Case Report of an Advanced Esophageal Neuroendocrine Carcinoma

**DOI:** 10.7759/cureus.53027

**Published:** 2024-01-26

**Authors:** Kamlesh Taori, Vijendra Kirnake, Parmeshwar R Junare, Vishal Padwale

**Affiliations:** 1 Department of Gastroenterology and Hepatology, Jawaharlal Nehru Medical College, Datta Meghe Institute of Medical Sciences, Wardha, IND; 2 Department of Gastroenterology, Jawaharlal Nehru Medical College, Datta Meghe Institute of Medical Sciences, Wardha, IND

**Keywords:** neuroendocrine tumor, small cell carcinoma, esophageal cancer, chemotherapy, case report

## Abstract

Esophageal neuroendocrine carcinomas (E-NECs) are rare malignant tumors with unknown etiology and pathogenesis. The aggressive nature of E-NECs coupled with a tendency to metastasize and no available treatment guidelines lead to poor prognosis. Here, we report a case of a 65-year-old, previously healthy female who presented with difficulty in swallowing solids, burning sensation over the epigastric region, weight loss (>10%), and altered bowel habits for the last three months. Contrast-enhanced CT (CECT) thorax revealed asymmetric mid-esophageal wall thickening with lymphadenopathy and metastasis in both hepatic lobes. Esophageal endoscopy revealed a large circumferential ulcero-proliferative mass and umbilicated lesions. A histopathological examination revealed small cells with scant cytoplasm and pleomorphic, hyperchromatic nuclei with prominent nuclear molding, and extensive necrosis. Immunohistochemistry revealed positivity for synaptophysin, chromogranin A, and CD56. Ki-67 index was 53%. These findings suggested poorly differentiated, small cell type, high-grade E-NEC. Chemotherapy with cisplatin (30mg/m^2^) + irinotecan (60mg/m^2^) was initiated. However, following two chemotherapy cycles, the patient succumbed.

## Introduction

Esophageal neuroendocrine carcinomas (E-NECs) are extremely rare malignant epithelial tumors with high neuroendocrine differentiation [1]. They mainly originate from the middle (55.1%) and lower (34.7%) esophageal segments [2]. Morphologically, neuroendocrine tumors (NETs) are either small cell (about 90%) or large cell (about 10%). With an incidence rate of 0.44 per million a year, they account for 0.04% of all NECs, and 0.4 - 2% of all esophageal cancers [3]. Over the last few decades, their incidence has increased, mainly owing to clinician awareness and improved diagnostic modalities. Most of the E-NECs have poor prognosis, mainly due to their aggressive nature, tendency to metastasize, and lack of standard treatment [1]. The available literature documents the incidence of E-NECs in other parts of the world [4]; however, E-NECs are rarely reported among Indian patients [5]. Herein, we report a case of an elderly woman with metastatic E-NEC who succumbed within two months of diagnosis.

## Case presentation

A 65-year-old, previously healthy female presented with difficulty in swallowing solids for the last three months. She had a history of burning sensation over the epigastric region, weight loss (>10%), and altered bowel habits. She had hypertension and diabetes mellitus for the last five years. There was no history of fever, vomiting, or abdominal trauma. There was an absence of any significant family history. Except for the presence of moderate anemia (hemoglobin 8 gm%), all other laboratory investigations, including complete blood count, coagulation profile, kidney function test, liver function test, and routine urine analysis, were within normal limits.

**Figure 1:**
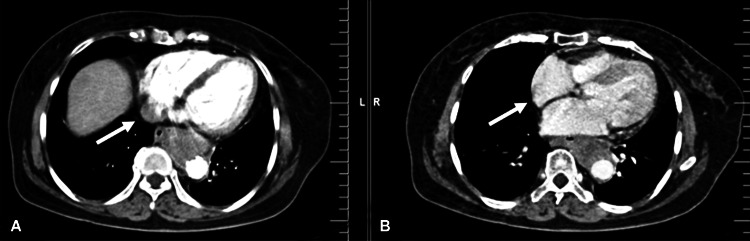
Contrast-enhanced CT of the thorax illustrating heterogeneously enhancing asymmetric wall thickening in mid-esophagus with extension (A) and lymphadenopathy (B).

Contrast-enhanced CT (CECT) of the thorax revealed heterogeneously enhancing asymmetric wall thickening (maximum 23mm) in the mid-esophagus with extension (15cm) and lymphadenopathy. Multiple hypo-enhancing round-to-oval lesions were observed in both hepatic lobes, with the largest lesion noted in the right lobe (2.5×2.3cm) (Figure 1). These findings were suggestive of esophageal lesion of malignant etiology with hepatic metastasis.

**Figure 2:**
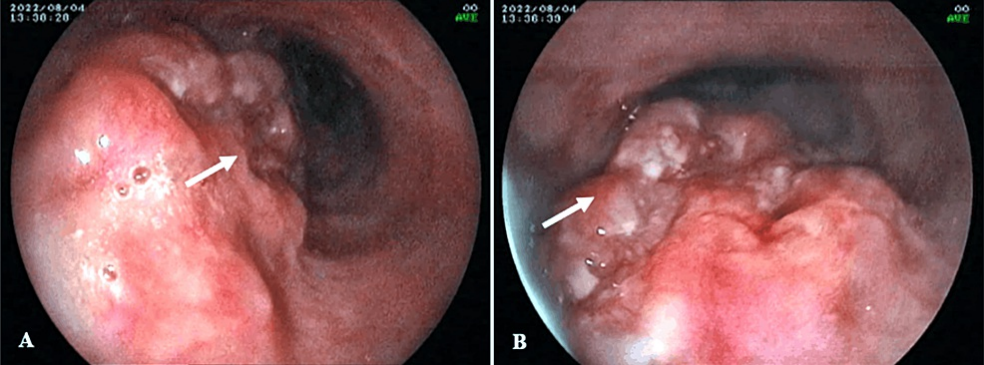
Upper gastrointestinal endoscopic examination illustrating a large circumferential ulceroproliferative friable mass in the esophagus (A and B).

Upper gastrointestinal endoscopic (UGIE) examination revealed a large circumferential ulceroproliferative friable mass at 26cm from the incisor and extending up to 30cm suggestive of esophageal growth. Moreover, there was the presence of two umbilicated lesions in the esophagus, at 24 and 34cm (Figure 2).

**Figure 3:**
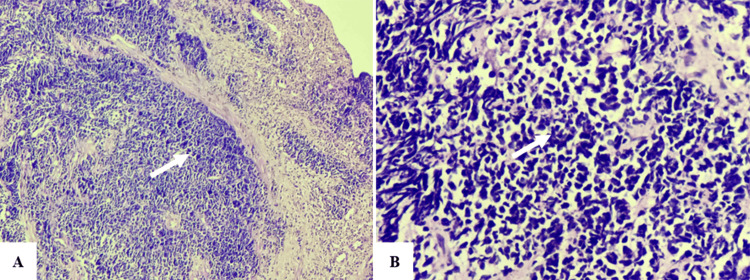
Histopathological examination illustrating an infiltrating malignant epithelial tumor consisting of small cells with scant cytoplasm and pleomorphic, hyperchromatic nuclei (10× H&E stain, A). The malignant nuclei showed prominent nuclear molding, and extensive necrosis suggestive of a poorly differentiated, small cell type E-NEC (40× H&E stain, B).

A histopathological examination of the upper gastrointestinal endoscopy (UGIE) specimen revealed an infiltrating malignant epithelial tumor consisting of small cells with scant cytoplasm and pleomorphic, hyperchromatic nuclei. The malignant nuclei showed prominent nuclear molding, and extensive necrosis was also noted. These findings led to the diagnosis of a poorly differentiated, small cell type E-NEC (Figure 3). On immunohistochemistry (IHC), the tumor was positive for synaptophysin, chromogranin A, and CD56. Additionally, the Ki-67 index was 53%. Thus, the high-grade nature of the E-NEC was confirmed.

Based on the diagnosis and assessment of the patient's status, surgical resection and radiotherapy of E-NEC were avoided, and chemotherapy with cisplatin (30mg/m2) + irinotecan (60mg/m2) every other week was initiated. However, following two chemotherapy cycles, the patient succumbed.

## Discussion

Florence McKeon, in 1952, initially reported two high-grade oat cell E-NECs. Thereafter, more than 4000 cases have been described. The incidences vary according to geographic location: 0.5-5.9%, 0.8-2.8%, and 1-2.8% of all esophageal cancers in China, Japan, and the Western world, respectively [4]. E-NECs mainly afflict elderly individuals (range 42-85 years), with a predilection for the male sex (80%) [3]. The symptoms' onset is 1-12 months prior to the diagnosis. This delay is mostly a result of non-specificity, absence, or delayed onset of the symptoms. However, the time to diagnosis is less than other histological types, mainly due to its aggressive nature [4].

E-NECs are mainly located in the middle-to-lower esophageal segments, due to the greater availability of Merkel cells and endocrine cells in the cardiac glands in the middle and lower esophagus, respectively. These malignant cells may be functional; however, most of them are non-functional, particularly the poorly differentiated ones [3]. E-NECs are usually an incidental finding on UGIE, with the visualization of a single lesion. However, the lesion may appear as an ulcerated or fungating mass, with deep infiltration of the esophageal wall [4]. The symptoms vary according to the size, location, secretory activity, and metastasis of the tumor [3]. Dysphagia and weight loss are the most common presenting symptoms, while dysphonia, odynophagia, retrosternal and epigastric pain, dyspnea, and gastrointestinal bleeding are also reported [1,4]. Similarly, our case was a female, aged 65 years, who had symptoms for three months and presented with dysphagia, retrosternal burning sensation, weight loss, and altered bowel habits. UGIE revealed a circumferential ulcero-proliferative mass with two umbilicated lesions located in the middle and lower esophageal segments. Additionally, the aggressive nature of the NEC, particularly the high-grade tumor, leads to a high proportion of metastasis during patient presentation [3]. Similarly, due to the high-grade tumor, our patient had metastasis in the regional lymph nodes and liver.

Though risk factors associated with E-NECs are not precisely known, tobacco smoking and alcohol intake are mainly implicated. Additionally, E-NECs are reported to be associated with a history of gastroesophageal reflux disease, achalasia, and Barrett’s metaplasia [3,4]. All these risk factors are linked to squamous cell carcinoma and adenocarcinoma of the esophagus. Contrarily, the patient in this report was previously healthy and had no history of tobacco smoking and alcohol intake with the absence of a family history of malignancy.

Only a small proportion of E-NECs are well-differentiated (<1%), while the rest are high-grade poorly differentiated small-cell E-NECs. The latter ones mostly have lymph nodes and distant metastases (31-90% cases). The targets for distant metastases are generally the lung, liver, and bone [1,4]. Similarly, our patient had high-grade poorly differentiated small-cell E-NECs. Microscopically, small-cell E-NECs comprise small ovoid, round, or spindle-like cells with hyperchromatic nuclei and dense chromatin associated with repeated mitoses and multifocal necrosis. While, large cell E-NECs, observed less frequently, include large cells with prominent nucleoli with marked nuclear atypia and basophilic cytoplasm. On IHC, synaptophysin, chromogranin A, and CD56 illustrate neuroendocrine differentiation, and their positivity ranges from 60% to 100% with chromogranin A expression, generally faint and/or focal [1]. Similarly, in this report, the E-NEC consisted of small cells with scant cytoplasm and pleomorphic, hyperchromatic nuclei with prominent nuclear molding, and extensive necrosis. Additionally, IHC revealed positivity for synaptophysin, chromogranin A, and CD56.

In patients with E-NEC, poor prognosis may be ascribed to rare incidence, non-specific presentation, and lack of dedicated tumor, nodes, and metastases (TNM) classification [2]. Additionally, there is a lack of consensus or guidelines for the treatment of E-NEC. However, various modalities have been reported, including endoscopic treatment, surgery, radiotherapy, chemotherapy, biological therapy, and targeted therapy [6]. When an E-NEC is 0.2-0.8cm in size and there is an absence of regional lymph node metastases, endoscopic treatment is suitable, while surgical excision of the tumor is appropriate for patients with primary tumors or those with regional lymph node metastasis [7]. A platinum-based chemotherapy regimen, involving cisplatin + irinotecan/etoposide is effective against E-NECs, with a good tolerability profile [4]. Due to locally advanced and metastatic disease at presentation, our case was not a candidate for surgical resection. As surgical resection was not performed, we could not comment on the TNM stage of the tumor. Thus, the case was initiated on cisplatin + irinotecan.

It is reported that, relative to patients with mixed E-NECs, those with pure E-NECs have significantly worse prognosis [2,4]. The survival time depends on prognostic factors, including age, limited or extensive disease, stage, and treatment type [8]. Depending on the limited or extensive nature of the disease, a median survival time of a maximum of 20 months or 6-12 months is reported, respectively. Our case had moderate anemia, >10% weight loss, and extensive disease with hepatic metastasis. Despite initiating chemotherapy, the patient could not survive beyond two months.

## Conclusions

E-NECs are extremely rare, and thus their etiology, pathogenesis, and prognosis remain to be determined. Findings suggest that elderly females presenting with a history of dysphagia and weight loss over months, even without exposure to risk factors such as smoking and alcohol, need to be suspected for E-NECs. Patients, especially those with poorly differentiated, small cell type E-NECs, present with extensive disease with metastasis. With their increasing incidence, prospective studies should be conducted, and evidence-based treatment guidelines need to be formulated.
